# Mortality and Non-Fatal Clinical Outcomes After the Most Common Cancers in People with HIV: A Multicohort Collaboration

**DOI:** 10.3390/cancers17244000

**Published:** 2025-12-16

**Authors:** Alisa Timiryasova, Lauren Greenberg, Pere Domingo, Philip E. Tarr, Alexander Egle, Charlotte Martin, Cristina Mussini, Ferdinand Wit, Antonella Cingolani, Clara Lehmann, Antonella Castagna, Kathy Petoumenos, Caroline A. Sabin, Fabrice Bonnet, Jens Lundgren, Martina Bottanelli, Sean Hosein, Christina Carlander, Alain Amstutz, Katharina Grabmeier-Pfistershammer, Harmony Garges, Andrea Marongiu, Lital A. Young, Lars Peters, Lene Ryom, on behalf of the D:A:D and RESPOND Study groups

**Affiliations:** 1 CHIP—Centre of Excellence for Health, Immunity and Infections, Rigshospitalet, University of Copenhagen, DK-2100 Copenhagen, Denmark; 2 HIV Infection Unit, Hospital de la Santa Creu I Sant Pau, Sant Quintí, 89, 08025 Barcelona, Spain; 3 Reial Acadèmia de Medicina de Catalunya, 08001 Barcelona, Spain; 4 Swiss HIV Cohort Study (SHCS), Kantonsspital Baselland, University of Basel, 4101 Bruderholz, Switzerland; 5 3rd Medical University Department, Paracelsus Medical University, 5020 Salzburg, Austria; 6 Infectious Diseases Department, CHU Saint-Pierre, Université Libre de Bruxelles, 322 rue Haute, 1000 Brussels, Belgium; 7 Modena HIV Cohort, Università Degli Studi Di Modena and Reggio Emilia, 41125 Modena, Italy; 8 AIDS Therapy Evaluation in the Netherlands (ATHENA) Cohort, Stichting HIV Monitoring & Department of Internal Medicine, Amsterdam UMC, University of Amsterdam, 1018 VN Amsterdam, The Netherlands; 9 Italian Cohort Naive Antiretrovirals (ICONA), Fondazione Policlinico A. Gemelli, IRCCS, L.go A. Gemelli 8, 00168 Roma, Italy; 10 Department I of Internal Medicine, Medical Faculty and University Hospital Cologne, University of Cologne, 50937 Cologne, Germany; 11 German Center for Infection Research (DZIF), Partner Site Bonn-Cologne, 50937 Cologne, Germany; 12 San Raffaele Scientific Institute, Università Vita-Salute San Raffaele, 20132 Milano, Italy; 13 The Australian HIV Observational Database (AHOD), Kirby Institute, University of New South Wales (UNSW), Sydney 2052, Australia; 14 Centre for Clinical Research, Epidemiology, Modelling and Evaluation, Institute for Global Health, University College London, London NW3 2PF, UK; 15 CHU de Bordeaux and Bordeaux University, BPH, INSERM U1219, 33076 Bordeaux, France; 16 European AIDS Treatment Group, 1000 Brussels, Belgium; 17 Department of Infectious Diseases, Karolinska University Hospital, 141 86 Stockholm, Sweden; 18 Division of Clinical Epidemiology, University Hospital Basel, 4051 Basel, Switzerland; 19 Electronic Health Records Group, Population Health Sciences, Bristol Medical School, University of Bristol, Bristol BS8 1QU, UK; 20 Oslo Centre for Biostatistics and Epidemiology, Oslo University Hospital, University of Oslo, 0372 Oslo, Norway; 21 Department of Dermatology, Medical University of Vienna, 1090 Vienna, Austria; 22 ViiV Healthcare, Global Medical, Durham, NC 27709, USA; 23 Real World Evidence Virology Department, Gilead Sciences, London WC1V 7EE, UK; 24 Global Medical Affair, Merck Sharp & Dohme, Rahway, NJ 07065, USA; 25 Department of Clinical Medicine, University of Copenhagen, DK-1165 Copenhagen, Denmark; 26 Department of Infectious Diseases, Hvidovre University Hospital, DK-2650 Copenhagen, Denmark

**Keywords:** cancer, HIV, mortality, non-fatal clinical outcomes, modifiable risk factors, immune status, comorbidities

## Abstract

**Background/Objectives**: Whilst cancer is a leading cause of death in people with HIV, less is known about clinical outcomes after cancer. **Methods**: Participants from the RESPOND and D:A:D cohorts with the five most common cancers (Kaposi’s sarcoma (KS); non-Hodgkin lymphoma (NHL); and lung, anal and prostate cancers) were followed from first cancer diagnosis after 2006/2012 [D:A:D/RESPOND] until death, final follow-up or administrative censoring (2016/2021). Incidence rates (IR) were calculated for post-cancer mortality; for non-fatal events (cardiovascular disease, diabetes, another primary cancer, AIDS events) individually and as a non-fatal composite clinical outcome (CCO). Predictors or mortality and CCO were assessed using Poisson regression with generalized estimating equations. **Results**: Amongst 2485 participants with cancer, mortality and CCO IRs were highest after lung cancer (445.4/1000 person years [95% CI 399.7, 494.9], 117.1 [94.3, 143.8], respectively) compared to other cancers and lowest after KS (21.3 [16.9, 26.6], 43.9 [37.5, 51.3]). The most common non-fatal outcomes were AIDS events after NHL and KS, diabetes after lung and prostate cancer and another primary cancer after anal cancer. Among people with NHL and anal cancer, a diagnosis in more recent years was associated with lower mortality risk. Increasing the time-updated CD4 count reduced mortality by 15–40% (per 100 cells/µL) after NHL and anal and lung cancers and reduced CCO risk by 17–28% after KS and NHL. Smoking, low BMI and multimorbidity increased CCO risks by two to three times after KS and NHL. **Conclusions**: Risk of post-cancer mortality and non-fatal outcomes varies by cancer type and risk profile, suggesting the need for personalized post-cancer clinical monitoring.

## Simple Summary

While cancer is a leading cause of death in people with HIV, less is known about clinical outcomes after cancer. We aimed to assess outcomes after the five most common cancers in people with HIV (Kaposi’s sarcoma (KS); non-Hodgkin lymphoma (NHL); and lung, anal and prostate cancers) in two international HIV cohort collaborations (D:A:D and RESPOND). We assessed incidence rates and risk factors of mortality, cardiovascular disease, diabetes, another primary cancer and AIDS events individually and as a non-fatal composite clinical outcome (CCO). Amongst 2485 participants, those with lung cancer had the highest rates of mortality, CCO and diabetes, whilst those with KS had the lowest rates of mortality, CCO, diabetes and cardiovascular disease. Higher CD4 count consistently protected against post-cancer mortality and CCO. Modifiable lifestyle factors (smoking, low BMI and high comorbidity burden) increased the risk of CCO after NHL and KS. These results highlight the importance of personalized post-cancer clinical monitoring.

## 1. Introduction

People with Human Immunodeficiency Virus (HIV) have a higher incidence of several cancers, including lung and anal cancers, as well as lymphomas [1,2]. This increased incidence is multifactorial and related to immunosuppression, higher coinfection rates with pro-oncogenic viruses and lifestyle factors such as smoking [3,4]. With widespread access to antiretroviral therapy (ART), there has been a large decline in the incidence of several cancers, including those previously termed Acquired Immune Deficiency Syndrome (AIDS)-defining cancers (Kaposi’s sarcoma (KS), non-Hodgkin lymphoma (NHL) and cervical cancer) and other infection-related cancers [5,6]. However, the incidence of other cancer types (e.g., smoking- and obesity-related cancers) has slightly increased [5].

Cancer has become a leading cause of death among people with HIV in high- and middle-income countries [6,7,8]. Additionally, some studies suggest that people with HIV and poor immune function, in particular, have higher mortality risk after cancer compared to the general population [9,10]. Beyond mortality, studies in the general population suggest that people with prior lung, breast and colorectal cancers are at increased risk of cardiovascular disease (CVD) compared to those without cancer, while data is more conflicting for other cancers, such as prostate cancer [11,12,13]. Similarly, other data from the general population suggest that people with cancer have increased risk of diabetes, often secondary to the cancer treatment [14,15,16,17]. Additionally, use of chemotherapy, corticosteroids and other immunosuppressive therapies may detrimentally lower immune function and increase the risk of opportunistic infections, prompting recommendations of prophylaxis in HIV [18,19,20].

Assessments of cancer-related outcomes in people with HIV are limited to date. However, a recent US cohort study did find that the risk of severe events, such as diabetes and myocardial infarction, was increased among those with HIV and certain cancers compared to those with HIV but without cancer [21]. However, the investigators did not assess the impact of HIV-associated factors and focused exclusively on what were historically classified as non-AIDS-defining cancers. Insights into incidence rates (IRs) and predictors of mortality and non-fatal clinical outcomes after cancer in people with HIV are crucial for the optimization of clinical monitoring and personalized post-cancer care.

Using combined data from two large international observational cohorts of well-characterized people with HIV, we aimed to assess the IRs and risk factors for mortality, CVD, diabetes, another primary cancer and AIDS events following diagnosis of the most common individual cancers.

## 2. Materials and Methods

### 2.1. Study Design

The Data Collection on Adverse events of Anti-HIV Drugs (D:A:D) and International Cohort Consortium of Infectious Diseases (RESPOND) cohorts are prospective multi-cohort collaborations of people with HIV from across Europe, Australia and the US. D:A:D (1999–2016) includes data from approximately 49,000 people with HIV across 11 cohorts, while RESPOND (2017–present) includes data from approximately 35,000 people from 17 cohorts, with some overlap in cohorts and participants. Detailed information on both studies is published elsewhere [22,23]. The studies use a similar methodology, collecting standardized data during routine clinical care. Systematic cancer collection began in 2006 in D:A:D and in 2017 in RESPOND, with retrospective cancer events collected back to 2012 in RESPOND and earlier if available. In addition to annual collection of demographic and clinical data, specific serious clinical events (including death, cancer and CVD) are collected in real time using study-specific case report forms and centrally validated against pre-defined algorithms by trained physicians [24,25,26]. A selection of all cancer events is subsequently reviewed by an external oncologist.

### 2.2. Study Population

We included all participants from D:A:D and RESPOND aged 18 years or older at study entry and diagnosed after cohort enrolment with one of the five most common cancers in the cohorts, excluding precancerous lesions, non-melanoma skin cancers and relapses or metastases from previous cancers. To ensure a sufficient number of clinical outcomes after each cancer, we focused on the most common cancers: NHL; KS; and lung, anal and prostate cancers.

Participants were excluded from RESPOND if they lacked CD4 count or viral load (VL) measurements within one year before or 12 weeks after baseline, as defined below, as in prior RESPOND analyses [5]. These criteria were not used in the D:A:D study and, therefore, were not applied to D:A:D participants in our primary analysis but were considered in a sensitivity analysis. Participants were excluded from RESPOND and D:A:D if they lacked gender/sex data. Individual RESPOND cohorts with potential under-reporting of cancer and other clinical outcomes were excluded. Participants enrolled in both cohorts were counted only once, as in prior combined analyses [5].

Baseline was defined as the date of first diagnosis of one of the five most common cancers after the latest of cohort enrolment and 2006/2012 (D:A:D/RESPOND).

Participants were followed from the time of incident cancer until the earliest of death, final follow-up (D:A:D: date of last visit plus 6 months; RESPOND: the latest of the most recent CD4 count, VL measurement, ART start date, drop-out date or date of death) or the administrative censoring date (D:A:D: 1 February 2016; RESPOND: 31 December 2021). Participants enrolled in both D:A:D and RESPOND were followed until the final RESPOND follow-up.

### 2.3. Outcome Definitions

The main outcomes of interest following cancer diagnosis were severe clinical events systematically captured in both consortiums and included death (using the coding of death in HIV (CoDe) methodology [26]), non-fatal CVD, diabetes, another primary cancer and a new AIDS-defining event. Additionally, these four non-fatal outcomes were combined into a non-fatal composite clinical outcome (non-fatal CCO), as low event rates did not allow for an adequate assessment of the risk factors for each outcome separately. CVD was defined as a composite of myocardial infarction, stroke and invasive cardiovascular procedures (coronary angioplasty or stenting, coronary bypass surgery and carotid endarterectomy). Diabetes was defined as two confirmed random blood glucose levels ≥ 11.1 mmol/L, a single HbA1c ≥ 6.5%, use of antidiabetic medication or a cohort-reported clinical diagnosis [25]. Another primary cancer was defined as a new distinct cancer of a type other than the baseline cancer, excluding non-melanoma skin cancers, precancerous lesions, relapses and metastases from previously diagnosed cancers. AIDS events were based on the criteria established by the Centers for Disease Control and Prevention [27].

### 2.4. Potential Risk Factors

The following variables were considered as potential risk factors for mortality and clinical outcomes: calendar year, age, gender/sex, HIV acquisition mode, ethnicity/race, geographical region, body mass index (BMI), smoking status, CD4 count, VL, ART experience, cancer stage at time of diagnosis, comorbidities (including CVD, chronic kidney disease (CKD), AIDS, diabetes, other cancers, hypertension and dyslipidemia) and comorbidity burden. Definitions of all variables are provided in the Table 1 footnotes.

### 2.5. Statistical Analysis

Crude IRs and 95% confidence intervals ( CIs) for mortality, CVD, diabetes, another primary cancer, AIDS events and non-fatal CCO were calculated per 1000 person years of follow-up (PYFU) after cancer diagnosis. We also examined individual causes of death after cancer.

Poisson regression models with generalized estimating equations and robust standard errors were used to investigate associations between risk factors for mortality and non-fatal CCO after each cancer. Log follow-up time was included as an offset. Each potential risk factor was initially assessed in univariable models. Age, gender/sex, ART status, calendar year, BMI and smoking status were included in the multivariable models a priori. Other factors were included only if they achieved *p* < 0.1 in univariable analysis. Cancer stage was assessed as a risk factor only for lung, anal and prostate cancers, as it was not systematically collected in D:A:D for NHL and KS.

The mortality regression models were adjusted for prior and time-updated comorbidities, including CVD, CKD, AIDS, diabetes, other cancers, hypertension and dyslipidemia. However, due to limited statistical power, in the non-fatal CCO models, we combined these comorbidities into a comorbidity burden variable.

Data on the type of cancer treatment were not systematically available in D:A:D and were therefore not included in the analysis.

Missing data for categorical variables were accounted for by including an unknown category in all regression models.

### 2.6. Sensitivity Analyses

Several sensitivity analyses were conducted: (1) using a Fine–Gray model to treat death as a competing risk for CCO, (2) assessment of risk factors for non-fatal CCO with AIDS events excluded from the definition, (3) including only centrally validated clinical events, (4) excluding participants with missing data for any variables included in the multivariable model, (5) excluding participants with prior serious clinical events (CVD, AIDS, diabetes or cancer) and (6) applying RESPOND exclusion criteria to D:A:D participants.

Analyses were performed using Stata/SE 18.0 (StataCorp LLC, College Station, TX, USA).

## 3. Results

**Figure 1:**
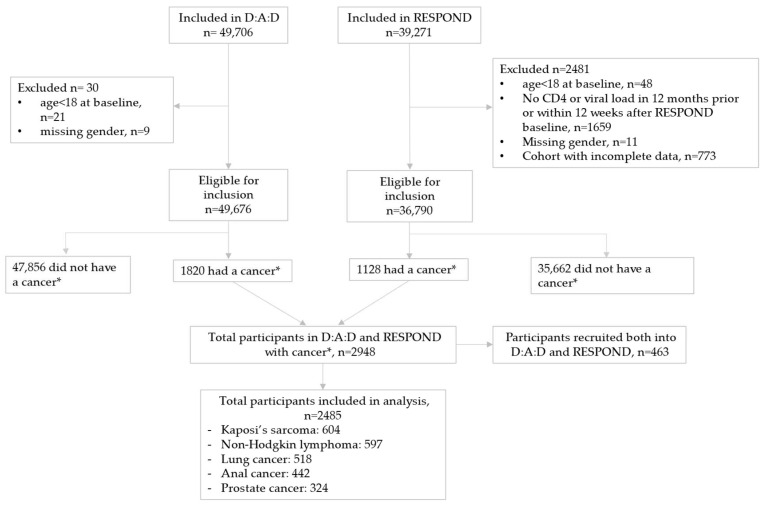
Study flow. * The five most common cancers in the combined D:A:D and RESPOND database: Kaposi’s sarcoma; non-Hodgkin lymphoma; and lung, anal and prostate cancers.

We included 1820 D:A:D and 1128 RESPOND participants with one of the five cancers. Of those, 463 participants were enrolled in both cohorts and only included once, resulting in 2485 unique participants (Figure 1). There were 604 (24%) participants with KS, 597 (24%) with NHL, 518 (21%) with lung cancer, 442 (18%) with anal cancer and 324 (13%) with prostate cancer.

### 3.1. Baseline Characteristics

Baseline characteristics (at cancer diagnosis) are presented in Table 1: 88% of participants were male, 56% were white, 14% were ART-naïve and 64% were virally suppressed. Median age was highest in those with prostate (64 years, interquartile range [IQR, 59–69]) and lung cancers (57 [51–63]) and lowest in those with KS (43 [36–51]). Whilst the majority of participants with lung cancer had disseminated disease at the time of diagnosis (58%), this was substantially less frequent for anal and prostate cancers (both 15%).

**Table 1:** Baseline characteristics (at cancer diagnosis) of participants overall and by cancer type.

	Overall (n = 2485)		Kaposi’s Sarcoma (n = 604)		Non-Hodgkin Lymphoma (n = 597)		Lung Cancer (n = 518)		Anal Cancer (n = 442)		Prostate Cancer (n = 324)		
	n	(%)	n	(%)	n	(%)	n	(%)	n	(%)	n	%	*p*
**Age, years**													<0.001
<40	430	(17.3)	242	(40.1)	142	(23.7)	9	(1.7)	38	(8.6)	0	(0.0)	
40–49	674	(27.1)	189	(31.2)	208	(34.7)	111	(21.4)	157	(35.5)	10	(3.1)	
50+	1381	(55.6)	173	(28.7)	247	(41.6)	398	(76.8)	247	(55.9)	314	(96.6)	
**Sex/Gender ^a^**													<0.001
Male	2190	(88.1)	559	(92.5)	503	(84.3)	414	(79.9)	390	(88.2)	324	(100.0)	
Female	294	(11.8)	44	(7.3)	94	(15.7)	104	(20.1)	52	(11.8)	0	(0.0)	
Transgender	1	(0.0)	1	(0.2)	0	(0.0)	0	(0.0)	0	(0.0)	0	(0.0)	
**Ethnicity/Race ^b^**													<0.001
White	1380	(55.5)	249	(41.0)	318	(53.4)	321	(62.0)	278	(62.9)	214	(66.6)	
Black	101	(4.1)	31	(5.1)	43	(7.2)	5	(1.0)	9	(2.0)	13	(4.0)	
Other	45	(1.8)	13	(2.2)	15	(2.5)	7	(1.4)	7	(1.6)	3	(0.9)	
Unknown	959	(38.6)	311	(51.7)	221	(36.9)	185	(35.7)	148	(33.5)	94	(29.9)	
**BMI, kg/m^2^**													<0.001
<18.5	156	(6.3)	33	(5.5)	33	(5.5)	53	(10.2)	29	(6.6)	8	(2.5)	
18.5–<25	1427	(57.4)	367	(60.6)	308	(51.8)	306	(59.1)	275	(62.2)	171	(52.8)	
>25	608	(24.5)	107	(17.8)	161	(26.9)	114	(22.0)	106	(24.0)	120	(37.0)	
Unknown	294	(11.8)	97	(16.1)	95	(15.9)	45	(8.7)	32	(7.2)	25	(7.7)	
**Geographical Region**													<0.001
Western Europe	1010	(40.6)	192	(31.6)	237	(39.9)	245	(47.3)	178	(40.3)	158	(48.8)	
Southern Europe	418	(16.8)	99	(16.4)	110	(18.4)	92	(17.8)	73	(16.5)	44	(13.6)	
Northern Europe	967	(38.9)	304	(50.5)	217	(36.2)	159	(30.7)	176	(39.8)	111	(34.3)	
Eastern Europe	79	(3.2)	9	(1.5)	24	(4.0)	20	(3.9)	15	(3.4)	11	(3.4)	
USA	10	(0.4)	0	(0.0)	9	(1.5)	1	(0.2)	0	(0.0)	0	(0.0)	
**HIV acquisition mode**													<0.001
MSM	1454	(58.5)	449	(74.3)	284	(47.7)	213	(41.1)	306	(69.2)	202	(62.3)	
IDU	271	(10.9)	11	(1.8)	79	(13.2)	124	(23.9)	45	(10.2)	12	(3.7)	
Heterosexual	597	(24.0)	109	(18.1)	178	(29.7)	152	(29.3)	67	(15.2)	91	(28.1)	
Other	50	(2.0)	6	(1.0)	16	(2.7)	12	(2.3)	7	(1.6)	9	(2.8)	
Unknown	113	(4.5)	29	(4.8)	40	(6.7)	17	(3.3)	17	(3.8)	10	(3.1)	
**ARV treatment history**													<0.001
Naive	344	(13.8)	208	(34.4)	95	(15.9)	18	(3.5)	13	(2.9)	11	(3.4)	
ART-experienced	2126	(85.6)	393	(65.1)	500	(83.8)	493	(95.2)	427	(96.6)	312	(96.3)	
**CD4 nadir ^c^, cells/µL**													<0.001
<200	1528	(61.5)	349	(57.8)	377	(63.3)	316	(61.0)	311	(70.4)	174	(53.7)	
200–350	601	(24.2)	146	(24.1)	143	(23.9)	136	(26.3)	75	(17.0)	102	(31.5)	
350–500	248	(10.0)	75	(12.5)	55	(9.2)	46	(8.9)	40	(9.0)	32	(9.9)	
>500	103	(4.1)	32	(5.3)	19	(3.2)	20	(3.9)	16	(3.6)	16	(4.9)	
**CD4, cells/µL**													<0.001
<100	312	(13.0)	154	(26.2)	115	(20.0)	20	(4.0)	19	(4.5)	4	(1.3)	
100–200	236	(9.9)	76	(13.0)	78	(13.5)	47	(9.4)	29	(6.9)	6	(1.9)	
200–350	477	(19.2)	122	(20.3)	139	(23.2)	101	(19.5)	76	(17.0)	39	(12.0)	
350–500	503	(20.2)	110	(18.3)	121	(20.2)	115	(22.2)	85	(19.2)	72	(22.2)	
>500	864	(34.8)	125	(20.8)	123	(20.5)	216	(41.7)	211	(47.7)	190	(58.6)	
**HIV VL, ^d^ copies/mL**													<0.001
<200	1600	(64.4)	176	(29.2)	317	(53.3)	438	(84.6)	371	(83.9)	296	(91.4)	
≥200	812	(32.7)	410	(67.8)	256	(42.7)	62	(12.0)	61	(13.8)	25	(7.7)	
Unknown	73	(2.9)	18	(3.0)	24	(4.0)	18	(3.5)	10	(2.3)	3	(0.9)	
**Smoking status**													<0.001
Never	533	(21.4)	187	(30.9)	156	(26.2)	18	(3.5)	71	(16.1)	101	(31.2)	
Current	964	(38.8)	174	(28.7)	203	(34.1)	300	(57.9)	208	(47.1)	79	(24.4)	
Previous	598	(24.1)	91	(15.1)	124	(20.7)	165	(31.9)	116	(26.2)	102	(31.5)	
Unknown	390	(15.7)	152	(25.2)	114	(19.0)	35	(6.8)	47	(10.6)	42	(13.0)	
**Diabetes ^e^**													<0.001
No	2204	(88.7)	553	(91.5)	532	(89.1)	451	(87.1)	395	(89.4)	273	(84.3)	
Yes	228	(9.2)	35	(5.8)	41	(6.8)	64	(12.4)	43	(9.7)	45	(13.9)	
Unknown	53	(2.1)	16	(2.7)	24	(4.0)	3	(0.6)	4	(0.9)	6	(1.9)	
**Prior AIDS**													<0.001
No	1564	(62.9)	439	(72.6)	377	(63.4)	310	(59.8)	218	(49.3)	219	(67.6)	
Yes	921	(37.1)	165	(27.4)	219	(36.6)	208	(40.2)	224	(50.7)	105	(32.4)	
**Prior NADC**													<0.001
No	2308	(92.9)	591	(97.8)	567	(95.0)	460	(88.8)	401	(90.7)	289	(89.2)	
Yes	177	(7.1)	13	(2.2)	30	(5.0)	58	(11.2)	41	(9.3)	35	(10.8)	
**Prior CVD ^f^**													<0.001
No	2306	(92.8)	580	(96.0)	576	(96.5)	459	(88.6)	407	(92.1)	284	(87.7)	
Yes	141	(5.7)	8	(1.3)	10	(1.7)	55	(10.6)	32	(7.2)	36	(11.1)	
Unknown	38	(1.5)	16	(2.7)	11	(1.8)	4	(0.8)	3	(0.7)	4	(1.2)	
**Comorbidity burden ^g^ (number of comorbidities)**													<0.001
0	389	(15.7)	194	(32.2)	112	(18.7)	37	(7.1)	28	(6.3)	18	(5.6)	
1	773	(31.1)	235	(38.7)	201	(38.6)	141	(27.2)	106	(24.0)	62	(19.1)	
2	711	(28.6)	115	(19.1)	163	(27.4)	157	(30.3)	160	(36.2)	115	(35.3)	
3+	612	(24.6)	60	(10.0)	92	(15.4)	183	(35.3)	148	(33.5)	129	(39.8)	
**Cancer stage ^h^**													<0.001
Localized	776	(31.2)	98	(16.1)	64	(10.7)	124	(23.9)	290	(65.6)	201	(62.0)	
Disseminated	567	(22.8)	52	(8.6)	105	(17.5)	298	(57.5)	64	(14.5)	48	(14.8)	
Unknown	1142	(46.0)	454	(75.2)	428	(71.8)	96	(18.5)	88	(19.9)	75	(23.1)	
**Cancer treatment**													<0.001
Chemotherapy	239	(9.6)	30	(4.9)	89	(14.9)	57	(11.0)	56	(12.7)	7	(2.2)	
Radiotherapy	145	(5.8)	5	(0.8)	7	(1.2)	40	(7.7)	58	(13.1)	35	(10.8)	
Surgery	125	(5.0)	8	(1.3)	3	(0.5)	38	(7.3)	33	(7.5)	43	(13.3)	
Endocrine therapy	26	(1.0)	0	(0.0)	1	(0.2)	1	(0.2)	0	(0.0)	24	(7.4)	
Immune therapy	36	(1.4)	2	(0.3)	18	(3.0)	15	(2.9)	0	(0.0)	1	(0.3)	
Antineoplastic therapy	22	(0.9)	3	(0.5)	11	(1.8)	2	(0.4)	2	(0.4)	4	(1.2)	
Unknown	1911	(76.9)	512	(84.7)	486	(81.5)	358	(69.1)	337	(76.2)	218	(67.3)	
	**Median**	**IQR**	**Median**	**IQR**	**Median**	**IQR**	**Median**	**IQR**	**Median**	**IQR**	**Median**	**IQR**	***p***
**Baseline date (month/year)**	12/11	(01/08, 07/15)	09/09	(03/07, 04/14)	04/10	(10/06, 08/14)	12/12	(04/09, 10/16)	04/12	(11/08, 11/15)	04/14	(11/10, 02/18)	<0.001
**Age, years**	52	(43, 61)	43	(36, 51)	48	(41, 56)	57	(51, 63)	52	(46, 58)	64	(59, 69)	
**CD4 nadir ^c^, cells/µL**	146	(49, 263)	160	(42, 290)	137	(49, 250)	150	(60, 247)	108	(26, 220)	180	(80, 285)	<0.001
**CD4, cells/µL**	399	(212, 600)	280	(90, 469)	300	(141, 465)	441	(281, 684)	502	(299, 718)	562	(430, 729)	<0.001
**VL ^d^, copies/mL**	50	(39, 3855)	18,410	(54, 152,600)	70	(50, 27,274)	50	(29, 50)	50	(39, 50)	40	(19, 50)	<0.001
**Duration of HIV years**	11.2	(3.8, 19.1)	3.6	(0.2, 9.8)	7.2	(2.1, 14.9)	16.4	(9.8, 22.0)	16.5	(10.7, 22.6)	16.1	(8.2, 22.7)	<0.001
**Duration on ART, years**	9.9	(3.2, 15.9)	1.9	(0.1, 10.3)	5.9	(1.0, 11.2)	12.9	(7.4, 17.8)	13.7	(8.8, 17.9)	13.6	(7.4, 18.9)	<0.001

Abbreviations: BMI, body mass index; MSM, men who have sex with men; IDU, injecting drug use; ARV, antiretroviral; ART, antiretroviral therapy; VL, viral load; AIDS, acquired immune deficiency syndrome; NADC, non-AIDS-defining cancer; IQR, interquartile range. Due to small numbers, Australia was combined with Northern Europe, and Eastern Central Europe was combined with Eastern Europe. ^a^ The mixture of sex and gender is collected. ^b^ The mixture of ethnicity/race is collected. Information on ethnicity/race is prohibited in several of the participating cohorts. ^c^ CD4 count was taken as the lowest CD4 count prior to baseline. If no CD4 count was measured, the first measurement within 12 weeks after baseline was used. ^d^ VL measurements below the level of detection but unknown reported lower limits of detection of the HIV-RNA assay were included to the category of < 200 copies/mL. ^e^ Diabetes was defined by a reported diagnosis, use of anti-diabetic medication, glucose ≥ 11.1 mmol/L, and/or HbA1c ≥ 6.5% or ≥48 mmol/mol. ^f^ Cardiovascular disease (CVD) was defined using a composite diagnosis of myocardial infarction, stroke or an invasive cardiovascular procedure. ^g^ Comorbidities included prior AIDS-defining and non-AIDS-defining cancers, AIDS events, chronic kidney disease (CKD), CVD, hypertension, diabetes and dyslipidemia. Hypertension was defined as two consecutive systolic blood pressure (SBP) measurements ≥ 140 mmHg and/or diastolic blood pressure (DBP) measurements ≥ 90 mmHg, performed on different days; one single SBP measurement ≥ 140 mmHg and/or DBP measurement ≥ 90 mmHg with the use of antihypertensive medication within six months of this measurement; or the initiation of antihypertensives without a recorded high BP reading. CKD was defined as confirmed if there were 2 consecutive measurements of estimated glomerular filtration rate (eGFR) ≤ 60 mL/min per 1.73 m^2^ measured at least 3 months apart or a 25% eGFR decrease when eGFR ≤ 60 mL/min per 1.73 m^2^. Dyslipidemia was defined as total cholesterol > 239.4 mg/dL, HDL cholesterol < 34.7 mg/dL, triglyceride > 203.55 mg/dL or use of lipid-lowering treatments. ^h^ Disseminated cancer stage defined as metastasized cancer. Cancer stage for NHL and KS was not collected in D:A:D.

### 3.2. Mortality After Cancer

**Figure 2:**
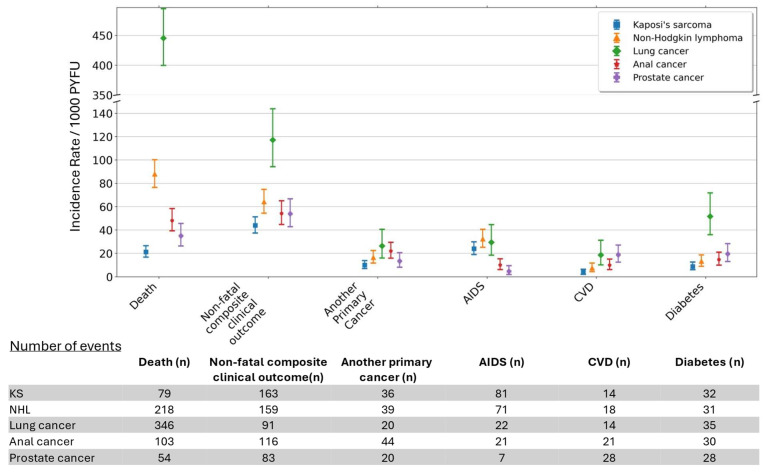
Crude Incidence Rates (IRs) per 1000 person years of follow-up for mortality and clinical events after cancer diagnosis. Non-fatal composite clinical outcome is a composite of another primary cancer, AIDS, CVD and diabetes. Abbreviations: AIDS, acquired immune deficiency syndrome; CVD, cardiovascular disease; KS, Kaposi’s sarcoma; NHL, non-Hodgkin lymphoma; PYFU, person years of follow-up.

Median follow-up after cancer overall was 3.1 years [IQR 0.8–7.1], with participants contributing a total of 10,630 PYFU. Participants with lung cancer had the shortest median follow-up time after cancer (0.7 years [IQR 0.3–1.7]), followed by those with NHL (2.5 [0.5–6.8]), anal cancer (4.0 [1.7–7.4]), prostate cancer (4.0 [1.7–6.8]) and KS (6.4 [2.9–8.8]), likely reflected in the differences in mortality after these cancers (Figure 2). Almost 21% of participants with cancer had died at one year after diagnosis (Table 2). The crude IRs of mortality varied by cancer type and were markedly higher after lung cancer (n = 346; 445.4/1000 PYFU [95% CI 399.7, 494.9]), with a 49% and 64% fatality rate at one and three years after diagnosis. Conversely, the lowest mortality incidence was seen after KS (n = 79; IR 21.3/1000 PYFU [16.9, 26.6]), with one- and three-year fatality rates of 8% and 11%.

Cancer was a leading underlying cause of death across all studied cancers, with 87% of all deaths after lung cancer, 72% after NHL, 52% after anal cancer, 41% after prostate cancer and 29% after KS. For KS and NHL, non-cancer AIDS events were also common causes of deaths (28% and 22%, respectively; Table A1).

**Table 2:** Cumulative percentage experiencing mortality and non-fatal clinical outcomes after the most common cancers by years of follow-up.

	Overall (%)	Kaposi’s Sarcoma (%)	Non-Hodgkin Lymphoma (%)	Lung Cancer (%)	Anal Cancer (%)	Prostate Cancer (%)
**1-year follow-up**						
Death	20.6	7.8	28.2	49.4	7.2	2.2
Non-fatal CCO	11.5	12.3	14	11.6	10	7.4
Cancer	3.1	2.2	3.5	3.1	4.5	2.2
AIDS	5.3	9.3	8.9	2.3	2	0.6
CVD	1.3	0.8	1	1.5	1.4	2.5
Diabetes	3.4	2.3	3.5	5	2.9	3.1
**2-year follow-up**						
Death	25.1	9.5	30.9	60	12	5.6
Non-fatal CCO	13.9	14	16	13	14.3	10.8
Cancer	4.1	3.3	4.5	3.5	6.3	3.1
AIDS	6.1	10	9.7	2.9	3.6	0.6
CVD	1.8	1	1.5	1.7	2.3	3.1
Diabetes	4.3	3.2	3.8	5.8	4.1	4.9
**3-year follow-up**						
Death	27.6	10.5	32.4	64.1	15.8	8.3
Non-fatal CCO	15.7	16.3	17	14.1	16.1	14.2
Cancer	4.7	4	4.7	3.7	7.2	4
AIDS	6.7	11.1	10.2	3.3	4.3	0.9
CVD	2.2	1.2	1.7	2.1	3.2	4
Diabetes	4.7	3.7	4	6.2	4.3	6.2

Abbreviations: non-fatal CCO, non-fatal composite clinical outcome; AIDS, acquired immune deficiency syndrome; CVD, cardiovascular disease.

### 3.3. Predictors of Mortality After Cancer

Risk factors for mortality following lung cancer, NHL and anal cancer are shown in a heat map in Table 3. Due to low mortality rates, we were underpowered to analyze risk factors for mortality after KS and prostate cancer.

After adjustment, mortality incidence declined over time by 7–10% per calendar year following NHL and anal cancer, respectively, while the decline for lung cancer was not significant after adjustment for other risk factors. Each increment of 10 years in age at cancer diagnosis was associated with 24% and 45% increased mortality for participants with NHL and anal cancer, respectively, but was not significantly associated with mortality following lung cancer. Mortality was also twofold higher for participants with disseminated anal cancer and nearly fivefold higher for those with disseminated lung cancer compared to participants with localized cancer of the same type. A higher time-updated CD4 count (per 100 cells/µL increment) was associated with reductions in mortality of 40%, 17% and 15% for NHL, anal cancer and lung cancer, respectively. Having another primary cancer during follow-up doubled the mortality risk after NHL and tripled it after anal cancer, whilst other comorbidities were not significant risk factors in our univariable model and, therefore, were not included in the multivariable model. Participants with injecting drug use (IDU) as the HIV acquisition mode had a threefold higher risk of anal cancer mortality compared to men who have sex with men (MSM), but this association was based on low numbers (20 events in persons with IDU and 61 MSM).

### 3.4. Non-Fatal Clinical Outcomes After Cancer

Overall, almost 12% of participants had a non-fatal CCO one year after cancer diagnosis, increasing to 16% by three years. The IR of non-fatal CCO after cancer was substantially higher for lung cancer (IR 117.1/1000 PYFU [95% CI 94.31, 143.82]) than for the other cancers considered (Figure 2).

The most common individual non-fatal clinical outcome following cancer depended on the cancer type. For NHL and KS, another AIDS event was most common (IR 32.9 [95% CI 25.21, 40.72] per 1000 PYFU and 24.0 [19.08, 29.87], respectively), with 9% of individuals with these cancers experiencing an AIDS event by one year. For lung and prostate cancers, the most common event was diabetes (IR 51.7 [36.01, 71.90] and 19.6 [13.04, 28.36], respectively), whereas for anal cancer, it was another primary cancer (22.0 [15.98, 29.53]), with 5% of individuals experiencing another cancer by 1 year (Table 2). CVD was one of the least common individual clinical outcomes after cancer, with just 1% and 2% of all participants experiencing such an event at one and three years.

### 3.5. Predictors of Non-Fatal Outcomes After Cancer

Predictors of non-fatal CCO after each cancer are shown in Table 3. Higher time-updated CD4 counts were associated with a 28% and 17% (per 100 cells/µL) reduced risk of non-fatal CCO after KS and NHL, respectively. For lung, anal and prostate cancers, similar trends were seen, but they did not reach statistical significance. When AIDS events were excluded from the non-fatal CCO, the beneficial effect of a higher CD4 count remained only for KS (aIRR 0.96, 95% CI [0.78, 0.96]; Supplementary Table S1). VL was not significant in univariable models (apart from for KS).

Older age (per 10 years) was associated with a 34% increased risk of non-fatal CCO after anal cancer. Smoking doubled non-fatal CCO risks after anal cancer, KS and NHL. Further, for participants with NHL and KS, low BMI (<18.5) and high comorbidity burden (>3 conditions) increased non-fatal CCO risk by 2–3-fold.

No statistically significant predictors of non-fatal CCO were identified after lung and prostate cancers, likely due to insufficient statistical power.

### 3.6. Sensitivity Analyses

All findings were largely consistent across all sensitivity analyses.

## 4. Discussion

This is the first large multinational study examining incidence and predictors of mortality and non-fatal clinical outcomes after the most common cancers in people with HIV. With an increasing proportion of aging people with HIV at risk of cancer, insights into predictors—particularly the role of potential modifiable risk factors—contributing to morbidity and mortality post cancer are increasingly pertinent to continuously improve HIV care. We found that post-cancer prognosis and predictors of clinical outcomes varied by cancer type. Our findings illustrate the need for a personalized and holistic post-cancer management strategy for people with HIV after a cancer diagnosis.

Participants with lung cancer commonly presented with disseminated disease and had the highest rate of mortality, with almost 50% dying within the first year after diagnosis. These findings are consistent with prior findings in people with HIV and in the general population [2,28,29]. As such, in the general population, mortality after a lung cancer diagnosis has 5-year relative survival rates of 16–27% compared to, e.g., prostate cancer, with 96% [30]. Furthermore, we found no indication of improved prognosis of lung cancer mortality over time, which highlights a pressing need for earlier diagnosis and continued efforts to intervene against modifiable risk factors for lung cancer, especially smoking cessation. While smoking status did not impact mortality incidence following lung cancer in our analysis, the number of persons without a smoking history was very limited. Smoking is generally considered a risk factor for increased mortality in the general population following a lung cancer diagnosis [31]; this effect is most clearly established at 5 and 10 years post diagnosis, and conversely, current data regarding 1-year survival rates remain inconsistent [32,33]. It is also possible that current and former smokers are diagnosed with lung cancer at an earlier stage than non-smokers due to increased screening within this high-risk group, potentially mitigating short-term mortality risks [32,34].

Participants with lung cancer had a high rate of non-fatal CCO. Although there were relatively few events compared to other cancers (likely because participants died before experiencing a non-fatal CCO), the number of events relative to the short follow-up time remained high.

The high incidence of diabetes, especially after lung cancer, likely reflects the common use of corticosteroids in this population, as also noted in previous studies, including a recent US analysis [14,21,35]. Diabetes incidence was also increased after prostate cancer, where the substantially lower mortality risk ensured a longer follow-up time at risk of non-fatal events and, as also noted in other studies, could be related to both treatment effects and shared risk factors [14,35,36,37]. These findings highlight the importance of monitoring blood glucose, especially after lung and prostate cancer.

The incidence of AIDS events was, as expected, increased after NHL and KS, reflecting the often-close connection to recently diagnosed HIV with high VL, low CD4 count and other viral coinfections. However, the incidence of AIDS was also notably increased after lung cancer, although affecting a smaller proportion of persons (2–3%) and likely related to both the cancer itself and to cancer treatment-related immunosuppression [18].

Participants with anal cancer predominantly had localized disease at the time of diagnosis and low mortality rates. With the longer follow-up time at risk of other non-fatal outcomes, another primary cancer was the most common event after anal cancer, as also described in a more recent register study and possibly explained by shared risk [38].

Prior studies in the general population and one study in population with HIV have described increased CVD rates after several cancers; however, in our study, CVD rates were fairly low over follow-up [11,12,13,21].

Among the strongest predictors for mortality were, as expected, disseminated disease for lung and anal cancer, whilst cancer stage did not reach statistical significance for non-fatal CCO after these cancers. Other strong mortality predictors were age and history of a prior (different) cancer for anal cancer and NHL. In contrast, age was not significantly associated with lung cancer mortality, potentially related to the high proportion with disseminated disease. Age only reached significance for non-fatal CCO after anal cancer, although the trend was in a similar direction for the other cancers.

Even in an aging population, mortality after NHL and anal cancer gradually improved over calendar time, likely reflecting, in part, effective and well-tolerated ART and improvements in cancer treatment regimens. Our follow-up precedes that of the ANCHOR study, demonstrating encouraging benefits of systematic screening for anal cancer and treatment of pre-cancer lesions [39].

Consistent with predominantly older studies focusing on cancer overall rather than individual cancers, we found that higher CD4 counts significantly decreased mortality rates after NHL, as well as after anal and lung cancers [40,41]; predictors of mortality were not assessed after KS and prostate cancer due to low mortality rates. Higher CD4 counts also predicted lower non-fatal CCO incidence after NHL and KS, although, particularly for NHL, this, to a large extent, was attributed to other AIDS events.

Similar but non-significant trends were seen for CCO after lung, anal and prostate cancers, also supporting the pathogenesis of these events in individuals without cancer [42,43]. Investigation of predictors of CD4 count trajectories after cancer is the focus of another ongoing project in RESPOND [44].

Consistent with our observation that cancer was the most common cause of death, multimorbidity did not substantially impact mortality risk. A different pattern was observed for non-fatal CCO; having >3 comorbidities substantially increased the incidence of non-fatal CCO, particularly after NHL and KS. Similar but non-significant trends were seen for anal and prostate cancers. Additionally, for participants with KS and NHL, other modifiable risk factors such as low BMI and a history of smoking also increased the risk of non-fatal CCO. Low BMI was predominantly associated with the cancers previously termed AIDS-defining cancers, likely serving as a proxy for overall frailty of individuals with AIDS as opposed to those experiencing a cancer without a similar degree of immunosuppression. Conversely, we observed no association between low BMI and CCO after cancers in participants with non-AIDS-defining cancers.

Our findings collectively underscore the need for a multidisciplinary approach to post-cancer care in people with HIV to address modifiable risk factors, including nutritional support, smoking cessation and proactive management of comorbidities.

Key strengths of our study include having a large and diverse study population across Europe, Australia and the US over extended calendar time, together with systematic and rigorous data collection and adjudication of clinical events. However, our study also has several limitations. Due to low numbers of non-fatal clinical events after cancers, we were unable to study predictors for these events individually. Additionally, even with the combined data from two of the largest longitudinal studies of people with HIV, the median follow-up time after cancer was relatively short; therefore, we may have underestimated the true disease burden after these cancers. Lack of data on cancer stage for some cancers (for D:A:D) and histological subtypes (RESPOND) prevented analysis of mortality differences between cancer subtypes. Missing data on specific cancer treatments, especially from D:A:D, precluded us from studying their impacts on participants’ death rates and other outcomes. Finally, data to further quantify tobacco usage, including pack years, was unfortunately not systematically available in D:A:D and RESPOND.

## 5. Conclusions

In this large multicohort study of people with HIV, rates and predictors of clinical outcomes after the most common cancers varied by cancer type. Lung cancer was often disseminated at the time of diagnosis and had the most unfavorable prognosis. Encouragingly, mortality after anal cancer and NHL declined over time. Among the strongest mortality predictors were disseminated cancer for lung and anal cancers and prior cancer and older age for NHL and anal cancer. Higher CD4 counts reduced rates of both fatal and non-fatal events after several cancers. Smoking, low BMI and multimorbidity increased incidence of non-fatal CCO, especially after KS and NHL. Our findings call for personalized and multidisciplinary post-cancer care strategies to improve long-term outcomes.

## Figures and Tables

**Table 3:** Heat map of Adjusted Incidence Rate Ratios (aIRRs) and 95% confidence intervals (95% CIs) for key predictors of mortality and non-fatal composite clinical outcome (CCO) after lung cancer, non-Hodgkin lymphoma (NHL), anal cancer, Kaposi’s sarcoma (KS) and prostate cancer *.

	Lung Cancer		Non-Hodgkin Lymphoma		Anal Cancer		Kaposi’s Sarcoma	Prostate Cancer
	Mortality	CCO	Mortality	CCO	Mortality	CCO	CCO	CCO
	aIRR (95%CI)	aIRR (95%CI)	aIRR (95%CI)	aIRR (95%CI)	aIRR (95%CI)	aIRR (95%CI)	aIRR (95%CI)	aIRR (95%CI)
Calendar year (per 1-year increase)	0.98 (0.95; 1.01)	1.00 (0.95; 1.06)	0.90 (0.86; 0.94)	0.96 (0.91; 1.02)	0.93 (0.89; 0.98)	0.95 (0.90; 1.00)	1.01 (0.96; 1.07)	0.99 (0.93; 1.06)
Age (per 10-year increase)	1.01 (0.86; 1.18)	1.12 (0.79; 1.60)	1.24 (1.03; 1.48)	1.10 (0.89; 1.38)	1.45 (1.13; 1.85)	1.34 (1.02; 1.76)	1.27 (1.00; 1.62)	1.29 (0.93; 1.80)
Female gender (ref.: male)	0.99 (0.70; 1.40)	0.77 (0.37; 1.61)	0.81 (0.49; 1.33)	0.57 (0.27; 1.20)	NA	NA	2.21 (1.02; 4.78)	NA
IDU as HIV acquisition mode (ref.: MSM)	NA	NA	NA	NA	3.06 (1.78; 5.26)	NA	0.72 (0.38; 1.36)	NA
CD4 increase per 100 cells/µL	0.85 (0.80; 0.90)	0.93 (0.83; 1.03)	0.60 (0.53; 0.68)	0.83 (0.73; 0.94)	0.83 (0.73; 0.94)	0.88 (0.78; 1.00)	0.72 (0.64; 0.81)	0.94 (0.84; 1.05)
Viral load at the baseline ≥ 200 copies/mL (ref.: <200)	NA	NA	NA	NA	NA	NA	0.82 (0.49; 1.35)	NA
BMI < 18.5 (ref.: 18.5–25.5 kg/m^2^)	1.29 (0.80; 2.08)	0.74 (0.32; 1.72)	1.24 (0.49; 3.15)	2.94 (1.43; 6.04)	1.00 (0.44; 2.30)	1.40 (0.65; 3.01)	3.63 (1.76; 7.48)	0.55 (0.08; 3.90)
BMI ≥ 25 (ref.: 18.5–25.5 kg/m^2^)	0.89 (0.62; 1.30)	0.96 (0.47; 1.96)	1.00 (0.67; 1.49)	0.79 (0.47; 1.34)	0.55 (0.30; 1.00)	0.89 (0.47; 1.68)	1.05 (0.62; 1.78)	1.10 (0.64; 1.90)
Current smoking (ref.: never)	1.07 (0.45; 2.56)	0.72 (0.16; 3.18)	1.52 (0.92; 2.51)	1.15 (0.59; 2.22)	0.70 (0.34; 1.47)	2.42 (1.03; 5.68)	1.99 (1.05; 3.79)	1.26 (0.65; 2.44)
Previous smoking (ref.: never)	0.88 (0.36; 2.14)	0.52 (0.12; 2.29)	1.07 (0.6; 1.92)	2.39 (1.27; 4.50)	1.08 (0.48; 2.43)	2.34 (0.96; 5.72)	0.78 (0.35; 1.70)	0.74 (0.38; 1.45)
Disseminated cancer stage (ref.: localized)	4.69 (3.27; 6.72)	1.25 (0.66; 2.37)	NA	NA	2.05 (1.24; 3.40)	1.24 (0.65; 2.38)	NA	0. 87 (0.4; 1.88)
Cancer *	1.60 (0.98; 2.59)	NA	2.35 (1.32; 4.20)	NA	3.50 (1.74; 7.04)	NA	NA	NA
Hypertension	NA	NA	1.15 (0.80; 1.67)	NA	NA	NA	NA	NA
Comorbidity burden ** (n = 1 (ref.: 0))	NA	NA	NA	0.73 (0.38; 1.38)	NA	1.92 (0.55; 6.71)	1.00 (0.55; 1.81)	0.75 (0.19; 3.01)
Comorbidity burden ** (n = 2 (ref.: 0))	NA	NA	NA	1.72 (0.87; 3.43)	NA	2.18 (0.64; 7.44)	1.09 (0.51; 2.29)	0.98 (0.27; 3.50)
Comorbidity burden ** (n = 3 (ref.: 0))	NA	NA	NA	2.34 (1.12; 4.90)	NA	2.98 (0.86; 10.36)	2.38 (1.03; 5.53)	1.73 (0.49; 6.14)
ART-experienced (ref.: ART-naïve)	0.99 (0.55; 1.77)	NA	1.85 (1.08; 3.17)	0.69 (0.37; 1.29)	NA	NA	0.9 (0.52; 1.57)	NA
	Significant protective factor							
	Non-significant protective factor							
	Non-significant risk factor							
	Significant risk factor							

* Predictors of mortality not assessed for KS and prostate cancer due to low numbers. Abbreviations: IDU, injecting drug use; MSM, men who have sex with men; ART, antiretroviral therapy. All models adjusted for age (fixed at baseline), gender/sex (fixed at baseline), ART status (fixed at baseline), BMI (fixed at baseline), calendar year (time-updated), smoking status (time-updated)—these risk factors were a priori included in the multivariable model. Other risk factors were included in the multivariable model based on their p-value in the univariable model (<0.1 for inclusion): CD4 per 100 cells/µL increase (time-updated) and cancer (time-updated) were included in non-Hodgkin, as well as lung and anal cancer mortality models; CD4 per 100 cells/µL increase (time-updated) was included in all five CCO models, hypertension (time-updated) was only included in the non-Hodgkin lymphoma mortality model; HIV transmission risk (fixed at baseline) was only included in the anal cancer mortality model; cancer stage (disseminated vs. localized, defined at baseline) was included in lung, anal and prostate cancer models. Number of comorbidities (fixed at baseline) was included in all CCO models except lung cancer; HIV acquisition risk (fixed at baseline) and viral load (fixed at baseline) were only included in Kaposi’s sarcoma model. Cancer stage was a non-significant risk factor for NHL in the univariable model and, therefore, was not included in the multivariable model. Predictors of mortality after KS and prostate cancer were not assessed due to low numbers and are not presented here. * Cancer—a different primary cancer prior the baseline or during the follow-up. ** Comorbidity burden defined at baseline, including prior AIDS-defining and non-AIDS-defining cancers, AIDS events, chronic kidney disease, cardiovascular disease, hypertension, diabetes and dyslipidemia.

## Data Availability

The RESPOND Scientific Steering Committee (SSC) encourages the submission of concepts for research projects. Online research concepts should be submitted to the RESPOND secretariat (respond.rigshospitalet@regionh.dk); for guidelines on how to submit research concepts, see the RESPOND governance and procedures point 6. The secretariat will direct the proposal to the relevant Scientific Interest Group, where the proposal will initially be discussed for scientific relevance before being submitted to the SSC for review. Once submitted to the SSC, the research concept’s scientific relevance, relevance to RESPOND’s ongoing scientific agenda, design, statistical power, feasibility and overlap with already approved projects will be assessed. Upon completion of the review, feedback will be provided to the proposer or proposers. In some circumstances, a revision of the concept might be requested. If the concept is approved for implementation, a writing group will be established, consisting of the proposers (up to seven people who were centrally involved in developing the concept), representatives from RESPOND cohorts and representatives from the Statistical Department and Coordinating Center. All individuals involved in the process of reviewing these research concepts are bound by confidentiality. All data within RESPOND from individual cohorts are de-identified. The present RESPOND data structure and a list of all collected variables and their definition can be found online. For any inquiries regarding data sharing, please contact the RESPOND secretariat (respond.rigshospitalet@regionh.dk).

## Supplementary Materials

The following supporting information can be downloaded at: https://www.mdpi.com/article/10.3390/cancers17244000/s1, Table S1: Heatmap of Adjusted Incidence Rate Ratios (aIRR) and 95% confidence intervals (95% CI) for key predictors of non-AIDS composite clinical outcome (CCO) after lung cancer, non-Hodgkin lymphoma (NHL), anal cancer, Kaposi’s sarcoma (KS) and prostate cancer.

## Abbreviations

The following abbreviations are used in this manuscript:

HIV	Human Immunodeficiency Virus
AIDS	Acquired Immune Deficiency Syndrome
ART	Antiretroviral therapy
KS	Kaposi’s sarcoma
NHL	Non-Hodgkin Lymphoma
IR	Incidence rate
CI	Confidence interval
CCO	Composite clinical outcome
CVD	Cardiovascular disease
VL	Viral load
BMI	Body mass index
CKD	Chronic kidney disease
PYFU	Person years of follow-up
IQR	Interquartile range
IDU	Injecting drug use
MSM	Men who have sex with men

## Appendix A

**Table A1:** Causes of death after all cancers, Kaposi’s sarcoma, non-Hodgkin lymphoma, lung cancer, anal cancer and prostate cancer.

	Overall, All Cancers (N = 800)		Kaposi’s Sarcoma (N = 79)		Non-Hodgkins Lymphoma (N = 218)		Lung Cancer (N = 346)		Anal Cancer (N = 103)		Prostate Cancer (N = 54)	
	n	(%)	n	(%)	n	(%)	n	(%)	n	(%)	n	(%)
**Cause of death**												
AIDS events (non-cancer)	51	(6.4)	22	(27.8)	24	(11.0)	2	(0.6)	3	(2.9)	0	(0.0)
AIDS malignancy	184	(23.0)	23	(29.1)	157	(72.0)	1	(0.3)	3	(2.9)	0	(0.0)
Infection	6	(0.8)	1	(1.3)	1	(0.5)	1	(0.3)	2	(1.9)	1	(1.9)
Bacterial infection	10	(1.2)	6	(7.6)	0	(0.0)	2	(0.6)	2	(1.9)	0	(0.0)
Pneumonia	1	(0.1)	0	(0.0)	0	(0.0)	0	(0.0)	0	(0.0)	1	(1.9)
HBV	1	(0.1)	0	(0.0)	1	(0.5)	0	(0.0)	0	(0.0)	0	(0.0)
HCV	6	(0.8)	2	(2.5)	2	(0.9)	1	(0.3)	1	(1.0)	0	(0.0)
Acute myeloid leukemia	1	(0.1)	0	(0.0)	1	(0.5)	0	(0.0)	0	(0.0)	0	(0.0)
Anal cancer	58	(7.2)	3	(3.8)	0	(0.0)	0	(0.0)	54	(52.4)	1	(1.9)
Bladder cancer	1	(0.1)	0	(0.0)	0	(0.0)	0	(0.0)	1	(1.0)	0	(0.0)
Chronic lymphoid leukemia	2	(0.2)	0	(0.0)	1	(0.5)	1	(0.3)	0	(0.0)	0	(0.0)
Connective tissue cancer	3	(0.4)	0	(0.0)	2	(0.9)	1	(0.3)	0	(0.0)	0	(0.0)
Esophagus cancer	1	(0.1)	0	(0.0)	1	(0.5)	0	(0.0)	0	(0.0)	0	(0.0)
Gallbladder cancer	1	(0.1)	0	(0.0)	0	(0.0)	0	(0.0)	1	(1.0)	0	(0.0)
Gynecologic cancer	1	(0.1)	0	(0.0)	1	(0.5)	0	(0.0)	0	(0.0)	0	(0.0)
Head and neck cancer	2	(0.2)	1	(1.3)	0	(0.0)	0	(0.0)	1	(1.0)	0	(0.0)
Hodgkin lymphoma	2	(0.2)	0	(0.0)	2	(0.9)	0	(0.0)	0	(0.0)	0	(0.0)
Liver cancer	1	(0.1)	0	(0.0)	1	(0.5)	0	(0.0)	0	(0.0)	0	(0.0)
Lung cancer	311	(38.9)	1	(1.3)	2	(0.9)	300	(86.7)	3	(2.9)	5	(9.3)
Metastasis of adenocarcinoma	2	(0.2)	0	(0.0)	0	(0.0)	2	(0.6)	0	(0.0)	0	(0.0)
Multiple myeloma	1	(0.1)	1	(1.3)	0	(0.0)	0	(0.0)	0	(0.0)	0	(0.0)
Pancreatic cancer	2	(0.2)	0	(0.0)	1	(0.5)	0	(0.0)	0	(0.0)	1	(1.9)
Prostate cancer	22	(2.8)	0	(0.0)	0	(0.0)	0	(0.0)	0	(0.0)	22	(40.7)
Rectum cancer	1	(0.1)	0	(0.0)	0	(0.0)	0	(0.0)	1	(1.0)	0	(0.0)
Stomach cancer	1	(0.1)	0	(0.0)	0	(0.0)	0	(0.0)	1	(1.0)	0	(0.0)
Unspecified leukemia	2	(0.2)	0	(0.0)	1	(0.5)	0	(0.0)	1	(1.0)	0	(0.0)
Unspecified Malignancy	30	(3.8)	4	(5.1)	4	(1.8)	16	(4.6)	5	(4.9)	1	(1.9)
Acute myocardial infarction	3	(0.4)	1	(1.3)	1	(0.5)	0	(0.0)	0	(0.0)	1	(1.9)
Heart or vascular disease	5	(0.6)	2	(2.5)	1	(0.5)	0	(0.0)	1	(1.0)	1	(1.9)
Myocardial Iinfarction or other ischemic heart disease	1	(0.1)	0	(0.0)	0	(0.0)	0	(0.0)	1	(1.0)	0	(0.0)
Lung embolus	1	(0.1)	0	(0.0)	0	(0.0)	1	(0.3)	0	(0.0)	0	(0.0)
Other ischemic heart disease	1	(0.1)	0	(0.0)	0	(0.0)	0	(0.0)	0	(0.0)	1	(1.9)
Stroke	8	(1.0)	1	(1.3)	2	(0.9)	0	(0.0)	1	(1.0)	4	(7.4)
Chronic obstructive lung disease	3	(0.4)	0	(0.0)	0	(0.0)	0	(0.0)	0	(0.0)	3	(5.6)
Chronic viral hepatitis	2	(0.2)	1	(1.3)	1	(0.5)	0	(0.0)	0	(0.0)	0	(0.0)
Degenerative disorders of the CNS	1	(0.1)	0	(0.0)	1	(0.5)	0	(0.0)	0	(0.0)	0	(0.0)
Digestive system disease	2	(0.2)	0	(0.0)	0	(0.0)	0	(0.0)	1	(1.0)	1	(1.9)
Hematological disease	1	(0.1)	1	(1.3)	0	(0.0)	0	(0.0)	0	(0.0)	0	(0.0)
Renal failure	2	(0.2)	1	(1.3)	1	(0.5)	0	(0.0)	0	(0.0)	0	(0.0)
Respiratory disease	2	(0.2)	0	(0.0)	1	(0.5)	0	(0.0)	1	(1.0)	0	(0.0)
Psychiatric disease	4	(0.5)	2	(2.5)	1	(0.5)	0	(0.0)	0	(0.0)	1	(1.9)
Mood or affective disorder	2	(0.2)	0	(0.0)	0	(0.0)	0	(0.0)	2	(1.9)	0	(0.0)
Accident or other violent death	1	(0.1)	1	(1.3)	0	(0.0)	0	(0.0)	0	(0.0)	0	(0.0)
Acute intoxication	1	(0.1)	0	(0.0)	0	(0.0)	0	(0.0)	1	(1.0)	0	(0.0)
Suicide	2	(0.2)	0	(0.0)	0	(0.0)	0	(0.0)	2	(1.9)	0	(0.0)
Other causes	1	(0.1)	0	(0.0)	0	(0.0)	0	(0.0)	0	(0.0)	1	(1.9)
Unknown	53	(6.6)	5	(6.3)	7	(3.2)	18	(5.2)	14	(13.6)	9	(16.7)

N—total number of deaths after each type of cancer.
