# Supplementary material for: Mortality and Non-Fatal Clinical Outcomes After the Most Common Cancers in People with HIV: A Multicohort Collaboration
Source: Cancers (Basel). 2025 Dec 16;17(24):4000. doi: 10.3390/cancers17244000 (PMC12731807; doi:10.3390/cancers17244000)
Supplement: Supplementary file 1 [file cancers-17-04000-s001.zip › cancers-3990272-supplementary.pdf]

**Supplementary Table S1.** Heatmap of Adjusted Incidence Rate Ratios (aIRR) and 95% confidence intervals (95% CI) for key predictors of non-AIDS composite clinical outcome (CCO) after lung cancer, non-Hodgkin lymphoma (NHL), anal cancer, Kaposi's sarcoma (KS) and prostate cancer.

| | Lung cancer non-AIDS CCO | NHL non-AIDS CCO | Anal cancer non-AIDS CCO | KS non-AIDS CCO | Prostate cancer non-AIDS CCO |
|---|---|---|---|---|---|
| | aIRR (95%CI) | aIRR (95%CI) | aIRR (95%CI) | aIRR (95%CI) | aIRR (95%CI) |
| Calendar year (per 1 year increase) | 1.01 (0.96; 1.08) | 1.01 (0.94; 1.08) | 0.94 (0.89; 1.00) | 1.09 (1.02; 1.18) | 0.99 (0.93; 1.06) |
| Age (per 10 years increase) | 1.15 (0.77; 1.70) | 1.19 (0.87; 1.61) | 1.44 (1.10; 1.89) | 1.46 (1.06; 2.02) | 1.38 (0.91; 1.79) |
| Female gender (ref.: male) | 0.62 (0.30; 1.28) | 0.32 (0.10; 0.99) | NA | 1.19 (0.31; 4.52) | NA |
| IDU as HIV acquisition mode (ref.: MSM) | NA | NA | NA | NA | NA |
| CD4 increase per 100 cells/µL | 0.94 (0.84; 1.05) | 0.93 (0.81; 1.05) | 0.94 (0.84; 1.06) | 0.86 (0.78; 0.96) | 0.94 (0.83; 1.06) |
| Viral load at the baseline ≥200 copies/mL (ref.:<200) | NA | NA | NA | NA | NA |
| BMI<18.5 (ref.: 18.5-25.5kg/m$^2$) | 0.64 (0.24; 1.73) | 3.08 (1.15; 8.28) | 0.84 (0.31; 2.24) | 4.31 (1.54; 12.09) | 0.60 (0.08; 4.35) |
| BMI≥25 (ref.: 18.5-25.5kg/m^2) | 1.26 (0.59; 2.66) | 1.00 (0.52; 1.92) | 1.07 (0.56; 2.07) | 1.63 (0.84; 3.15) | 1.20 (0.69; 2.10) |
| Current smoking (ref.: never) | 0.61 (0.13; 2.85) | 1.61 (0.70; 3.74) | 2.40 (0.94; 6.12) | 2.17 (0.64; 2.66) | 1.14 (0.58; 2.26) |
| Previous smoking (ref.: never) | 0.41 (0.08; 2.01) | 2.68 (1.18; 6.12) | 2.99 (1.14; 7.80) | 0.62 (0.15; 1.44) | 0.74 (0.37; 1.48) |
| Disseminated cancer stage (ref.: localised) | NA | NA | NA | NA | NA |
| Cancer* | NA | NA | NA | NA | NA |
| Hypertension | NA | NA | NA | NA | NA |
| Comorbidity burden** (n=1 (ref.:0)) | NA | 1.01 (0.41; 2.50) | 2.32 (0.52; 10.35) | 0.84 (0.39; 1.79) | 1.96 (0.23; 4.05) |
| Comorbidity burden** (n=2 (ref.:0)) | NA | 2.30 (0.88; 6.00) | 2.86 (0.66; 12.43) | 1.23 (0.45; 3.38) | 1.16 (0.31; 4.38) |
| Comorbidity burden** (n=3 (ref.:0)) | NA | 3.93 (1.39; 11.12) | 3.67 (0.83; 16.31) | 4.02 (1.42; 11.39) | 2.06 (0.55; 7.67) |
| ART-experienced (ref.: ART-naïve) | NA | 1.23 (0.46; 3.27) | NA | 0.76 (0.36; 1.61) | NA |

Significant protective factor
Non-significant protective factor
Non-significant risk factor
Significant risk factor

Abbreviations: IDU – injecting drug use, MSM – men who have sex with men, ART – antiretroviral therapy
All models adjusted for age (fixed at baseline), gender/sex (fixed at baseline), ART status (fixed at baseline), BMI (fixed at baseline), calendar year (time-updated), smoking status (time-updated) – these risk factors were a priori included in the multivariable model. Other risk factors were included in the multivariable model based on their p-value in univariable model (< 0.1 for inclusion).
*Cancer - a different primary cancer during follow-up
**Comorbidity burden defined at baseline, includes prior AIDS-defining and non-AIDS defining cancers, AIDS events, chronic kidney disease, cardiovascular disease, hypertension, diabetes, dyslipidemia
